# The Evolving Role of Radiation Therapy Technologists in Head and Neck Cancer: A Narrative Review and Operational Framework

**DOI:** 10.3390/jimaging12030117

**Published:** 2026-03-10

**Authors:** Andrea Lastrucci, Ilaria Morelli, Nicola Iosca, Isacco Desideri, Eva Serventi, Yannick Wandael, Carlotta Becherini, Viola Salvestrini, Vittorio Miele, Renzo Ricci, Lorenzo Livi, Pierluigi Bonomo, Daniele Giansanti

**Affiliations:** 1 Department of Allied Health Professions, Azienda Ospedaliero-Universitaria Careggi, 50134 Florence, Italy; 2 Radiation Oncology Unit, Azienda Ospedaliero-Universitaria Careggi, 50134 Florence, Italy; 3 Oncology Unit, Santa Maria delle Croci Hospital-AUSL Romagna, 48121 Ravenna, Italy; 4 Department of Medical Sciences (DIMEC), University of Bologna, 40126 Bologna, Italy; 5 Department of Experimental and Clinical Biomedical Sciences “M. Serio”, University of Florence, 50134 Florence, Italy; 6 Department of Allied Health Professions, Azienda USL Toscana Centro, 59100 Prato, Italy; 7 Department of Experimental and Clinical Biomedical Sciences, Emergency Radiology Unit, University of Florence, 50134 Florence, Italy; 8 Centre IATIS, ISS, 00161 Rome, Italy

**Keywords:** head and neck neoplasms, radiation therapy, patient-centered care, multidisciplinary care team

## Abstract

Head and neck cancer (HNC) management requires highly coordinated multidisciplinary care. Radiation Therapy Technologists (RTTs) have increasingly expanded their role beyond technical execution, contributing to patient positioning, treatment delivery, monitoring, and supportive care. This narrative review integrates evidence from published literature with structured experiential insights collected through focus group discussions with RTTs and other multidisciplinary team (MDT) members. The resulting conceptual and operational framework highlights RTT contributions across the radiotherapy pathway, including adaptive planning, workflow coordination, and patient-centered interventions, supported by imaging and artificial intelligence (AI) tools for predictive modeling and treatment optimization. By facilitating communication, monitoring anatomical and functional changes, and integrating AI-informed insights, RTTs support timely interventions, reduce treatment interruptions, and enhance treatment safety and precision. Structured training, formal recognition of advanced practice roles, and interprofessional collaboration are key to maximizing the impact of RTTs in HNC care. This review provides a practical roadmap for institutions, professional societies, and research initiatives to support the evolution of RTT roles in complex radiotherapy settings.

## 1. Introduction

### 1.1. Background

Head and neck cancer (HNC) represents a heterogeneous group of malignancies, accounting for approximately 5% of all neoplasms worldwide [1,2]. These tumors can originate from multiple anatomical sites within the head and neck region, including the nasopharynx, oral cavity, oropharynx, hypopharynx, larynx, sinonasal sinuses, and both major and minor salivary glands [2]. The diversity of anatomical subsites and histologies contributes to the complexity of HNC management and affects prognosis, treatment planning, and therapeutic outcomes. A substantial proportion of HNC patients present with locally advanced disease at diagnosis [3], often requiring multimodal treatment strategies, including surgery, radiotherapy, and systemic therapies.

A central component of modern HNC management is the head and neck multidisciplinary team (HN MDT), in which diagnostic, therapeutic, and supportive decisions are discussed collaboratively. MDTs are widely recognized as essential for optimizing treatment plans and ensuring comprehensive, coordinated care, with evidence suggesting potential improvements in clinical outcomes [4]. Through coordinated interaction among specialists, MDTs facilitate continuity of care and promote structured, guideline-based decision-making, supporting personalized strategies based on disease stage, comorbidities, and functional status [4,5,6]. MDT involvement is also associated with better adherence to guidelines, improved documentation, and enhanced patient satisfaction.

The effectiveness of MDTs relies on collaboration among diverse healthcare professionals, including Medical Oncologists, Radiation Oncologists, Surgeons, Radiologists, and Nursing staff. Among these, Radiation Therapy Technologists (RTTs) play a unique role as continuous points of contact for patients undergoing radiotherapy, bridging technical delivery and patient-centered care [7]. RTTs perform precise patient positioning, treatment delivery according to prescribed plans, plan verification, and monitoring of acute treatment-related effects. They contribute to imaging acquisition and position verification processes within image-guided radiotherapy workflows [8,9,10,11]. Standardized guidelines, such as those provided by ESTRO, further ensure safe and reproducible patient positioning and verification [12].

Despite their essential role, RTTs’ formal integration into MDT discussions remains inconsistent. Many teams rely predominantly on physicians for decision-making, while RTT insights—rooted in direct patient observation, workflow management, and imaging interpretation—are underutilized. Including RTTs in MDTs can strengthen clinical decision-making through real-time feedback on treatment tolerability, workflow feasibility, and early detection of side effects, potentially reducing interruptions and enhancing overall care quality.

### 1.2. Purpose

This paper presents a narrative review of the literature on RTTs in HN MDTs, complemented by structured expert input derived from multidisciplinary focus group discussions and clinical experience. It does not report original patient-level data. Using this approach, it synthesizes evidence and expert observations to propose a conceptual and operational framework, illustrating RTT contributions to treatment accuracy, imaging workflow management, toxicity monitoring, and patient-centered care. The study also addresses barriers to formal RTT integration, including variability in training, limited recognition, and organizational constraints, while proposing practical, literature- and experience-informed strategies to maximize RTT impact within multidisciplinary teams.

## 2. Study Design

This work adopts a narrative review approach, integrating evidence from published literature with structured experiential insights from RTTs in HN MDTs. No original quantitative data were collected; all observations are experience- and practice-informed.

The methodology combined two complementary components:

**Narrative literature review:** A structured search was performed using key terms related to head and neck cancer, Radiation Therapy Technologists, multidisciplinary teams, and imaging/AI-assisted workflows. Selection of studies followed the ANDJ checklist (CL) for narrative reviews, ensuring transparency and relevance. Included studies were further validated through a consensus-based report, provided as Supplementary Materials, which synthesizes and confirms the findings across multiple publications.

**Experience-informed observations through focus groups:** Insights were collected via structured reflection and discussions with RTTs and multidisciplinary stakeholders in dedicated focus group meetings. Participants were selected to ensure a **representative sample** of professionals across roles (RTTs, Radiation Oncologists, Medical Physicists, and Nursing staff) and institutions. Each focus group included multidisciplinary focal points that represented the collective experience of their entire clinical–technical units, typically encompassing 20–40 professionals per unit, ensuring a broad and representative perspective across institutions and professional societies.

These sessions captured practical experiences on workflow optimization, patient monitoring, and the integration of advanced imaging and AI tools. All observations are descriptive and illustrative, based on professional expertise rather than quantitative measurement.

The consensus-based report (provided as Supplementary Materials) functioned as a validation step of the framework. It served as a structured “cross-check” to verify alignment between literature-derived concepts (including studies from the narrative review and those cited in the Introduction) and practice-informed insights from the focus groups, ensuring internal coherence, clinical plausibility, and transferability across settings. No quantitative data was collected; all contributions are descriptive and grounded in professional expertise.

Key areas explored included:

- The optimization of immobilization devices, positioning reproducibility, and patient comfort.
- Patient care and support, including pre-treatment counseling, toxicity monitoring, and psychosocial support.
- Treatment scheduling and workflow coordination, including imaging acquisition, adaptive planning, and timely interventions.
- Active participation in MDT briefings, emphasizing RTT contributions to decision-making, interprofessional collaboration, and patient-centered care.

This design allows the integration of published evidence and structured professional experience to highlight best practices, challenges, and opportunities for RTT professional development. By combining the narrative review with validated experiential observations from representative focus groups, the study supports the development of an operational framework for RTT integration in HN MDTs without relying on original quantitative data.

## 3. Results

A total of 63 publications [1,2,3,4,5,6,7,8,9,10,11,12,13,14,15,16,17,18,19,20,21,22,23,24,25,26,27,28,29,30,31,32,33,34,35,36,37,38,39,40,41,42,43,44,45,46,47,48,49,50,51,52,53,54,55,56,57,58,59,60,61,62,63] were identified as relevant to the role of RTTs in HN MDTs. These works were functional to the study and contributed directly to the construction of the operational framework, in accordance with the study methodology. They provided context, evidence, and corroboration for the practice- and experience-informed observations collected through focus groups, without generating original quantitative data. No original quantitative data were collected; all insights are descriptive and grounded in professional expertise.

### 3.1. Strategic Roles and Contributions of RTTs in Head and Neck MDTs: Impact, Opportunities, and Emerging Recommendations

*RTTs’ impact on workflow and patient care*

RTTs represent frontline professionals in radiotherapy delivery and patient management [23,25,41], and their expertise optimizes multiple facets of HNC management. Drawing from a four-year experience at an Italian oncology center, RTT contributions extend beyond routine radiotherapy delivery to encompass patient care coordination, proactive monitoring, the integration of advanced imaging data, and the early adoption of AI-assisted decision support. Their involvement can significantly impact treatment quality, workflow efficiency, and patient-centered care.

Patient immobilization is critical to ensure reproducibility and precision in HNC radiotherapy, forming the foundation for accurate treatment planning and dose delivery [12,13]. The anatomical complexity of the head and neck region—steep dose gradients, proximity to critical structures, and frequent interactional variations—makes even minimal deviations in patient positioning clinically significant [8,9,11]. Standard immobilization devices, particularly closed-face thermoplastic masks, have long been the gold standard; however, they can cause patient discomfort, anxiety, and claustrophobia that potentially compromises treatment tolerance and positional stability, in some cases contributing to treatment session disruption [13,14,15,16,17].

Technological innovations and advanced imaging integration have enabled alternative immobilization solutions, including open-face masks and hybrid systems that balance comfort and rigidity. Pre-treatment CT, MRI, and PET-CT imaging, combined with daily Image-Guided Radiation Therapy (IGRT) verification, support individualized immobilization strategies and enable early identification of positioning variability during treatment [12,18]. Future AI-assisted imaging analytics may further enhance these decisions by predicting patient-specific movement patterns and optimal fixation strategies. Emerging AI-based imaging and radiomics tools are being investigated to support adaptive workflows and predictive modeling in head and neck radiotherapy [48,49,50,51,59,60,61,62]. This anticipatory involvement minimizes downstream delays in CT simulation, treatment planning, or radiotherapy initiation; enhances workflow efficiency; ensures reproducible immobilization across fractions; and enables more reliable IGRT alignment, ultimately improving treatment accuracy and safety.

Beyond technical responsibilities, RTTs play a pivotal role in patient care and support, particularly for those undergoing combined chemoradiotherapy, which is associated with higher toxicity, symptom burden, and unplanned interruptions [20,21,22]. RTTs provide patient education, psychosocial support, and the early identification of treatment-related complications, often informed by imaging and IGRT feedback [23,24,25]. Structured pre-treatment sessions prepare patients for technical, physical, and emotional aspects of radiotherapy, including explanations of immobilization procedures, expected toxicities, and supportive care strategies. Throughout therapy, RTTs monitor acute toxicities and leverage imaging feedback to detect subtle changes such as shifts in tissue geometry or weight loss. This information bridges nursing, medical, nutritional, and psychological teams, enabling early interventions that may help reduce unplanned treatment interruptions, which are associated with poorer oncologic outcomes [25,26,29]. RTTs also provide reassurance, motivational support, and practical counseling, reinforcing patient adherence and continuity of care. This expanded role is consistent with evidence supporting advanced practice RTT roles in toxicity assessment and adaptive care pathways [25,38,41,44]. They contribute to documenting treatment summaries, patient-reported outcomes, and adherence to surveillance schedules, strengthening continuity of care, ensuring timely multidisciplinary referrals, and facilitating longitudinal patient support.

RTTs also play a critical role in treatment scheduling and workflow coordination. Delays in radiotherapy initiation and prolongation of overall treatment time negatively affect local control and survival in HNC patients [26,29,30,31]. RTTs ensure that diagnostic imaging, simulation, contouring, planning, and verification occur on schedule. The integration of advanced imaging (CT, MRI, PET-CT) and daily IGRT data enables the proactive identification of bottlenecks and adaptation of treatment plans. RTTs liaise with physicists, clinicians, nutritionists, and nurses to ensure that imaging sequences, immobilization devices, and planning data are ready for timely execution. Future AI-assisted scheduling tools may allow the predictive modeling of workflow constraints, optimizing machine utilization and reducing treatment delays. Emerging AI-based auto-segmentation and radiomics tools are increasingly integrated into adaptive radiotherapy workflows in head and neck cancer [48,49,50,59]. Through active engagement in MDT meetings, RTTs integrate imaging information to streamline workflow, supporting adherence to guideline-recommended timelines.

Finally, RTTs contribute to multidisciplinary briefings, bridging daily technical operations, imaging insights, and broader objectives. HN radiotherapy is complicated by rapid anatomical changes, including tumor shrinkage, edema, mucosal inflammation, and weight loss, which may necessitate adaptive planning [33,34,35,36,45,46], increasingly supported by AI-assisted contouring and predictive modeling tools [48,49,50]. RTTs, observing patients at every fraction, detect these changes early using both technical assessment during patient positioning and imaging feedback from IGRT and other modalities [19,23,37]. Functional deterioration (dysphagia, aspiration risk, nutritional compromise) further motivates the integration of patient-specific imaging into MDT decision-making. Structured multidisciplinary pathways informed by imaging improve workflow, patient outcomes, and the early detection of adverse effects [39]. Weekly MDT briefings allow RTTs, together with Radiation Oncologists, to report relevant patient data—including side effects, weight loss, positioning issues, and imaging observations—supporting adaptive radiotherapy decisions. Future AI-based analytics could augment this process by predicting anatomical and functional changes, enabling more personalized, pathway-driven interventions. AI-driven radiomics and predictive modeling approaches are progressively reshaping personalization strategies in head and neck radiotherapy, supporting biologically informed and adaptive treatment paradigms [57,58,59,60,61,62].

To synthesize these complex roles, Table 1 provides a comprehensive overview of RTT contributions in HN MDT meetings, structured to show, for each domain, the key role, specific activities performed by RTTs, and the impact on MDT workflow and patient care. Table 2 provides a strategic, evidence-informed framework of recommendations for RTT integration, aligning strategic areas with actionable recommendations, literature justification, and expected impact on MDT workflow, AI utilization, and patient-centered outcomes [12,13,18,23,39,41,42].

This experience-informed exploration confirms that RTTs are far more than technical operators; their expertise spans the entire radiotherapy pathway—from immobilization optimization, patient monitoring, workflow coordination, adaptive planning, and imaging acquisition to the integration of advanced technologies including AI—substantially enhancing patient safety, therapeutic precision, continuity of care, and overall treatment quality [12,13,18,23,39].

*Emerging recommendations*

Table 2 provides a strategic, evidence-informed framework of recommendations for RTT integration, aligning strategic areas with actionable recommendations, literature justification, and expected impact on MDT workflow, AI utilization, and patient-centered outcomes [12,13,18,23,39,41,42]. 

*Key Challenges and Emerging Opportunities*

RTTs have the potential to significantly enhance HN MDT effectiveness, but their integration poses practical, educational, and organizational challenges. The expanding RTT responsibilities now require expertise beyond traditional technical functions, including advanced imaging, AI-assisted planning, and adaptive radiotherapy workflows [12,13,14,15,16,17,18,19,23,41]. Structured training programs, international guideline alignment, and periodic competency assessments are essential to maintain safe, effective, and evidence-based contributions [12,13,14,15,16,17,18,19,23,41].

The standardization of RTT roles remains critical. Variable or informal integration can lead to inconsistencies, task duplication, and communication gaps, potentially compromising care continuity and safety [42]. Institutional protocols and MDT endorsement are necessary to clarify responsibilities, strengthen interprofessional trust, and ensure accountability.

Technological evolution further complicates integration. Rapid advances in AI-driven imaging, adaptive radiotherapy, and real-time workflows require continuous RTT upskilling. Institutions must provide structured training, protected time, and competency-based credentialing to maintain proficiency and workflow efficiency [41].

Despite these challenges, RTT involvement offers substantial opportunities. Close patient interaction combined with expertise in imaging and AI enables timely interventions, early detection of toxicities, and patient-centered, adaptive care models [12,13,14,15,16,17,18,19,23,25,41]. RTTs also optimize workflow by coordinating imaging, simulation, and treatment phases, reducing delays, minimizing machine conflicts, and ensuring adherence to guideline-recommended timelines [30,31].

The adoption of AI-assisted tools, adaptive radiotherapy, and advanced imaging allows RTTs to integrate innovative practices into routine care, enhancing dose precision, anticipating anatomical changes, and supporting personalized treatments. This strengthens their technical expertise, career pathways, and the collaborative potential of MDT-led care.

Table 3 summarizes these challenges and opportunities, linking each item to its impact on MDT workflow, patient care, and professional development, complementing Table 1 on key RTT roles and translating experience into actionable insights.

### 3.2. Framework for RTT Integration in HN MDTs

The integration of RTTs within HN MDTs can be understood as a **three-level, continuum-based framework**, linking operational experience, emerging challenges, and strategic recommendations.

1. Operational Roles and Daily Activities (Table 1)
  - RTTs contribute directly to immobilization, patient care, treatment scheduling, and multidisciplinary briefings.
  - This level captures the day-to-day impact on workflow efficiency, treatment accuracy, and patient-centered care.
  - Examples: optimizing mask design, monitoring acute toxicities, coordinating imaging and simulation, reporting during MDT meetings.
2. Challenges and Opportunities (Table 3)
  - RTTs face **practical, educational, and technological challenges**, including rapid AI adoption, adaptive radiotherapy, and role standardization.
  - This level identifies systemic gaps and opportunities, linking routine activities to organizational needs and professional development.
  - Examples: unclear responsibilities, workflow bottlenecks, the need for continuous training, adoption of validated AI tools.
3. Strategic Recommendations (Table 2)
  - Insights from Table 1 and Table 3 inform **actionable, evidence-based strategies** to optimize RTT contributions.
  - Recommendations align with clinical justification, MDT workflow impact, AI integration, and patient-centered outcomes.
  - Examples: competency-based training programs, standardized protocols, structured AI-assisted planning, workflow optimization, interprofessional collaboration.

**Framework Logic (Textual Flow)**
  - Input → Daily Activities: Table 1 captures what RTTs do and their immediate impact.
  - Analysis → Challenges and Opportunities: Table 3 identifies gaps, emerging needs, and areas for professional growth.
  - Output → Recommendations: Table 2 proposes actionable strategies to enhance RTT integration, workflow efficiency, and patient care.

This textual framework provides a clear, sequential logic from descriptive practice to analytical insight to strategic implementation, without relying on a figure. It is adaptable, accommodating evolving technologies, AI tools, and institutional protocols [12,13,14,15,16,17,18,19,23,39,40,41,42].

## 4. Discussion

The evolving role of RTTs in HN cancer patients reflects the increasing complexity of multidisciplinary care and rapid technological innovation. This discussion synthesizes four years of experience at an Italian oncology center, highlighting how RTTs integrate technical expertise with advanced imaging and AI tools. We present a conceptual and operational framework for RTT contributions, illustrate their role in interprofessional collaboration, and outline future directions to enhance patient-centered care and professional development. These insights provide actionable guidance for institutions, professional societies, and research initiatives.

### 4.1. Evolving Role of RTTs in Multidisciplinary Teams: A Conceptual and Operational Framework

The management of HN cancer patients requires highly coordinated, multidisciplinary care. Over recent years, RTTs have evolved their role and now actively contribute to patient-centered care, workflow optimization, and adaptive interventions informed by advanced imaging and AI-assisted planning [25,43].

The study shows that RTT contributions span the entire radiotherapy pathway:

- Immobilization optimization and alignment with patient-specific anatomical considerations [12,13,18].
- Treatment scheduling and workflow coordination, ensuring timely imaging, simulation, and plan delivery [30,31,36].
- Real-time toxicity monitoring and patient support, enabling early interventions to prevent treatment interruptions [19,23,25,37].
- Adaptive planning and integration of imaging and AI data, supporting precision radiotherapy and personalized care [32,38,41,42,45,48].

By presenting RTT contributions as a continuous, integrated process rather than isolated tasks, this study proposes a conceptual and operational framework for RTT integration into HN MDTs. This framework is organized around:

1. Operational roles and daily activities (Table 1): detailing specific RTT tasks and their immediate impact on workflow and patient care.
2. Challenges and emerging opportunities (Table 3): identifying practical, educational, and technological factors affecting RTT practice.
3. Strategic recommendations (Table 2): translating experience and evidence into actionable guidance for training, role standardization, workflow optimization, AI integration, and patient-centered outcomes.

This structured approach allows institutions to implement RTT contributions systematically, supporting their professional development, interprofessional collaboration, and safe adoption of advanced technologies.

### 4.2. Integrating AI and Imaging: Current Practice vs. Future Potential

RTTs increasingly use AI and imaging tools to enhance workflow, adaptive planning, and toxicity management. It is essential to distinguish applications currently implemented in clinical practice from emerging or experimental tools.

*Current clinical applications:*

- Daily IGRT-based patient monitoring for positioning and anatomical changes [12,13,18,41]. RTTs review imaging data daily to ensure accurate alignment, detect deviations, and communicate adjustments to the planning team.
- AI-assisted auto-contouring for standard anatomical targets [48,49,50,53,54,55]. RTTs validate contours, identify inconsistencies, and ensure that automated delineation aligns with patient anatomy, preventing geometric errors that could compromise treatment.
- Predictive toxicity modeling integrated into planning workflow, with validated clinical evidence [56,57,59]. RTTs monitor patient-specific risk predictions for mucositis, xerostomia, or dysphagia, providing early alerts to the clinical team for intervention.

*Emerging or potential applications:*

- AI-driven adaptive dose optimization for individual tumor subregions [48,52,58]. RTTs may review model outputs to flag deviations from clinical targets or unexpected dose heterogeneity.
- Predictive modeling of inter-fractional anatomical and functional changes [51,59]. RTTs could integrate patient imaging trends with AI predictions to inform adaptive replanning decisions.
- Fully automated workflow scheduling with real-time adjustments [48,52,60]. RTTs may supervise automated prioritization and machine allocation, ensuring patient safety and protocol adherence.

RTTs are essential for validating AI outputs, contextualizing insights, and integrating them into MDT decisions, ensuring that interventions remain clinically safe, feasible, and patient-centered. This approach moderates forward-looking statements, grounding discussion in published evidence while highlighting areas that require further clinical validation. By actively engaging with both current and emerging AI tools, RTTs support precision, efficiency, and safety in HN radiotherapy, bridging technological innovation with practical patient care [41,48,57,59].

### 4.3. Comparison with International Frameworks and ESTRO Advanced Practice

The proposed framework was developed to describe and systematize RTT contributions in HN radiotherapy, based on four years of clinical experience. To contextualize its relevance, we compared it with existing international RTT role definitions and the ESTRO Advanced Practice (AP) frameworks [7,12,19,23,24] (Table 4).

This comparison illustrates how the proposed framework aligns with international guidance while offering additional specificity for HN radiotherapy. It emphasizes practical operationalization, making explicit the link between daily RTT tasks, challenges encountered, and actionable strategies. In particular, it:

- Integrates AI and advanced imaging into routine workflow and MDT discussions, distinguishing validated clinical applications from experimental tools.
- Highlights active MDT engagement, showing how RTTs can contribute to adaptive planning, toxicity monitoring, and workflow optimization in ways not explicitly detailed in current ESTRO guidance.
- Provides an evidence-informed roadmap that can support institutions in training, role standardization, and the systematic evaluation of RTT contributions.

By situating our framework alongside ESTRO and other international standards, it becomes clear where HN-specific operational guidance and AI-informed practices can complement and extend existing frameworks, offering a more comprehensive approach for modern radiotherapy teams.

### 4.4. Novel Contributions and Clinical Relevance

This study provides key insights into the evolving role of RTTs in head and neck radiotherapy, grounded in a narrative review of published literature complemented by structured experiential insights collected through multidisciplinary focus groups. A consensus-based report (CR) was used as a validation tool to ensure alignment between literature evidence and practice-informed observations, enhancing the framework’s credibility and transferability.

The primary contribution is an HN-specific operational framework that systematically maps RTT tasks across the radiotherapy workflow and links each activity to its impact on patient care, MDT efficiency, and workflow optimization.

Additional strengths of the framework include:

- Providing an evidence-informed roadmap that integrates daily operational tasks, practical challenges, and strategic recommendations, offering institutions actionable guidance for structured RTT integration.
- Formalizing RTT engagement in MDT processes, detailing their role in adaptive planning, imaging interpretation, and toxicity monitoring, thereby highlighting contributions to patient safety, treatment precision, and coordinated care.
- Contextualizing emerging AI applications within a practical framework, clearly distinguishing tools already implemented in routine practice from those requiring further validation, without overemphasizing speculative future technologies.

Overall, this work organizes dispersed literature and structured clinical experience into a coherent, transferable model for RTT practice in HN oncology, supporting professional development, optimizing clinical workflows, and guiding the systematic implementation of advanced RTT roles.

### 4.5. Future Directions

#### 4.5.1. Near-Term Operational Developments

In the near term, the role of RTTs is expected to expand in both operational and technological dimensions. A key priority is the systematic evaluation of procedural outcomes, including treatment efficiency, adherence to guideline-recommended timelines, toxicity management, adaptive replanning, and the minimization of treatment interruptions [25,41,43,44,47,53]. Such quantitative assessment will provide objective evidence of RTT contributions to workflow optimization and patient care.

The safe and effective integration of AI tools represents another immediate focus. Validated predictive models, AI-assisted auto-contouring, and workflow optimization tools [48,49,50,51,52] have begun to complement routine practice; however, structured training and competency assessment remain essential to ensure that these technologies are used safely and consistently [48,49,50,51,52]. RTTs play a critical role in interpreting AI outputs, contextualizing predictions, and integrating these insights into MDT decision-making.

Standardized education and simulation-based training are also crucial for preparing RTTs for complex clinical scenarios. Incorporating AI-imaging scenarios, adaptive planning exercises, and high-fidelity simulations enables RTTs to develop both technical proficiency and decision-making skills [54] in a controlled, educational environment [25,41,54].

Finally, enhanced MDT performance can be achieved by systematically quantifying RTT contributions to team efficiency, the speed and accuracy of adaptive planning, and overall patient outcomes [25,43,53]. Collectively, these near-term developments provide a foundation for the safe, effective, and evidence-based expansion of RTT responsibilities within head and neck radiotherapy workflows.

#### 4.5.2. Long-Term Precision Radiotherapy

Looking further ahead, the management of head and neck cancers increasingly requires precision approaches that account for biological heterogeneity across tumors. The integration of advanced imaging, radiomics, and AI-driven analytics will allow individualized dose distributions and adaptive treatment strategies tailored to tumor subregions, patient anatomy, and dynamic treatment responses [55,56,57,58,59,60,61,62].

In this context, RTTs will be central to implementing these precision approaches through:

- Advanced imaging acquisition and quality assurance—ensuring high-quality, reproducible imaging to support geometric, functional, and radiomic analyses.
- Active participation in ART workflows—monitoring anatomical and functional changes during treatment and flagging deviations that trigger adaptive replanning.
- Patient-centered monitoring for early toxicity detection and intervention—identifying subtle functional or anatomical changes to maintain safety and treatment continuity.

By combining technical expertise, experiential knowledge, and interprofessional collaboration, RTTs will ensure that emerging precision technologies are safely and effectively translated into routine clinical practice. Their role will be pivotal in delivering personalized, adaptive radiotherapy, maximizing therapeutic outcomes while minimizing toxicity, and reinforcing the integration of data-driven innovation into patient-centered care [54,55,56,57,58,59,60,61,62]. Moreover, future precision strategies will need to consider the optimal combination and sequencing of systemic therapies with radiotherapy to achieve synergistic effects and maximize treatment outcomes [63].

## 5. Conclusions

In this narrative review, we present a conceptual and operational framework for the integration of RTTs into head and neck cancer multidisciplinary teams, synthesizing four years of clinical experience and evidence from the literature. The framework highlights RTT contributions across the full radiotherapy pathway—from patient immobilization and treatment scheduling to real-time toxicity monitoring, adaptive planning, and the integration of advanced imaging and validated AI tools—enhancing treatment precision, safety, and patient-centered care.

By actively engaging in MDTs, RTTs strengthen interprofessional collaboration, streamline workflow, and ensure continuity of care, positioning themselves as pivotal facilitators of data-informed, patient-centered radiotherapy. The framework also identifies challenges, links them to operational and educational strategies, and provides actionable recommendations for training, role standardization, and the safe adoption of emerging technologies.

Looking ahead, the continued expansion of RTT responsibilities will require structured, competency-based education, standardized curricula, and guidance from professional societies to support advanced practice roles. RTTs are uniquely positioned to bridge clinical practice, technological innovation, and patient-centered care, facilitating the safe implementation of AI, adaptive radiotherapy, and precision approaches into routine practice. Through this narrative review and proposed framework, we provide practical guidance for institutions, educators, and policymakers aiming to optimize MDT performance, enhance professional development, and ultimately improve outcomes for head and neck cancer patients.

## Figures and Tables

**Table 1:** Key operational roles and contributions of RTTs in HN MDT meetings.

Domain	Key Role	Specific Activities	Impact on MDT Workflow and Patient Care
Immobilization Optimization	Ensure reproducible and precise patient positioning	Design and adjust immobilization devices (closed-face, open-face, hybrid masks) Integrate pre-treatment imaging (CT, MRI, PET-CT) and daily IGRT verification [12,13,14,15,16,17,18]	Minimizes simulation and planning delays Ensures reproducible positioning across fractions Improves IGRT protocols acquisition
Patient Care and Support	Educate, counsel, monitor, and support patients	Structured pre-treatment sessions; daily monitoring of acute toxicities (mucositis, dermatitis, dysphagia, xerostomia, fatigue) [20,21,22,23,24,25] Communication with multidisciplinary team	Early identification of complications Prevents treatment interruptions; strengthens continuity of care and patient adherence
Treatment Scheduling	Coordinate pre-treatment and therapy workflow	Ensure imaging, simulation, contouring, and verification occur on schedule Liaise with physicists, clinicians, nutritionists, nurses [30,31] Identify workflow bottlenecks	Reduces treatment delays; optimizes machine utilization Supports adherence to guideline-recommended timelines
Participation in Multidisciplinary Briefings	Integrate technical, imaging, and other data into MDT decisions	Report patient status, side effects, weight changes, positioning issues Support adaptive radiotherapy decisions [19,23,32,33,34,35,36,37]	Enhances MDT responsiveness; facilitates adaptive planning Improves patient outcomes through timely interventions

**Table 2:** Recommendations for enhancing RTT integration within HN MDTs.

Strategic Area	Key Recommendation	Key Impact on MDT and Patient Care	References
Structured Training and Continuous Education	Competency-based HN RT, advanced imaging (CT, MRI, PET-CT, IGRT), adaptive planning, AI-assisted decision support, patient-centered care; simulation-based learning and ongoing assessment	Prepares RTTs for MDT contributions, anticipates anatomical variations and treatment toxicities, reduces workflow variability	[12,13,14,15,16,17,18,20,21,22,23,24,25,41]
Role Standardization and Protocol Development	Define responsibilities and professional boundaries; standardized protocols for MDT participation, toxicity monitoring, patient education, adaptive planning	Ensures continuity of care, reduces ambiguity, strengthens accountability and interprofessional trust	[23,24,25,32,33,34,35,36,42]
Active Participation in MDT Decision-Making	RTTs present observations on immobilization, daily treatment variations, adaptive planning, patient monitoring, imaging acquisition	Enhances real-time problem-solving, adaptive plan implementation, treatment accuracy, and patient-centered outcomes	[12,13,14,15,16,17,18,19,23,32,33,34,35,36,37,41]
Workflow Optimization and Care Coordination	Coordinate imaging, simulation, treatment; use AI-assisted predictive scheduling	Minimizes delays, reduces bottlenecks, optimizes machine utilization, ensures guideline-adherent timelines	[26,27,28,29,30,31,41]
Integration of Technological Innovations	Adopt AI-assisted contouring, adaptive RT, automated imaging workflows; structured competency frameworks	Improves treatment precision, personalized care, reduces human error, supports MDT confidence in technology	[41,50,61]
Interprofessional Collaboration and Knowledge Sharing	Joint MDT rounds, shared imaging and AI review, collaborative problem-solving	Strengthens teamwork, holistic care, early detection of complications, and patient-centered outcomes	[23,24,25,37,38,41]
Monitoring and Evaluation of Outcomes	Longitudinal tracking of RTT contributions, workflow efficiency, re-planning triggers, patient outcomes	Informs continuous quality improvement, validates advanced roles, ensures safe, responsive care	[26,27,28,29,30,31,39,41]

**Table 3:** Key challenges and emerging opportunities for RTTs in head and neck radiotherapy MDT meetings.

Challenge/Opportunity	Description	Key Impact on MDT and Patient Care	References
Advanced Technical Expertise	RTTs require skills beyond traditional roles: AI-assisted planning, adaptive RT, imaging integration	Ensures millimeter-accurate treatment, supports biologically informed and adaptive strategies	[12,13,14,15,16,17,18,19,23,41]
Structured Training and Continuous Education	Progressive training, guideline alignment, workshops, periodic assessment	Maintains proficiency, reduces errors, strengthens interprofessional trust	[12,13,14,15,16,17,18,19,23,25,41]
Standardization of RTT Roles	Informal integration or unclear responsibilities can cause duplication, gaps, inconsistencies	Clarifies responsibilities, ensures continuity and safety, fosters MDT confidence	[42]
Technological Evolution	Rapid AI developments (auto-contouring, predictive analytics, real-time imaging)	Supports adaptive RT, anticipates anatomical/functional changes, enables innovative practice	[39,41]
Patient-Centered Interventions	Monitoring acute/late toxicities, proactive referrals, integration of imaging/AI	Prevents interruptions, improves adherence, enables personalized interventions	[12,13,14,15,16,17,18,19,23,25,41]
Workflow Optimization	Integration of procedural, logistical, imaging, and AI data to streamline RT	Reduces machine overload, avoids conflicts, ensures guideline-adherent timelines	[30,31]
Leadership and Role Recognition	Clear protocols, formal acknowledgment of RTT contributions	Promotes professional growth, active MDT participation, shared responsibility	[23,24,25,37,38]
Adoption of Innovative Technologies	AI-assisted contouring, predictive modeling, real-time imaging	Enhances precision, anticipates anatomical changes, supports personalized care	[39,41]

**Table 4:** Comparison of the proposed RTT integration framework with ESTRO advanced practice and international role definitions.

Feature	ESTRO AP Framework	Proposed Framework	Novelty/Extension
Core RTT technical tasks	Immobilization, treatment delivery, basic imaging	Same + adaptive planning, AI-assisted decision support	Incorporates HN-specific requirements, MDT integration, and predictive analytics for adaptive workflows
MDT participation	Limited mention	Active contribution to planning, toxicity monitoring, workflow optimization	Highlights interprofessional collaboration and real-time problem solving within MDTs
AI integration	Mentioned as future potential	Differentiates validated vs. emerging applications	Grounded in four-year clinical experience; specifies which tools are routinely used vs. experimental
Strategic guidance	Curriculum and training suggested	Evidence-informed recommendations for workflow, AI, patient-centered care	Provides an operational roadmap linking tasks → challenges → strategic recommendations, supporting systematic implementation

## Data Availability

No new data were created or analyzed in this study.

## Supplementary Materials

The following supporting information can be downloaded at https://www.mdpi.com/article/10.3390/jimaging12030117/s1, Table S1: Consensus report.
