# Supplementary material for: The Evolving Role of Radiation Therapy Technologists in Head and Neck Cancer: A Narrative Review and Operational Framework
Source: J Imaging. 2026 Mar 10;12(3):117. doi: 10.3390/jimaging12030117 (PMC13028032; doi:10.3390/jimaging12030117)
Supplement: Supplementary file 1 [file jimaging-12-00117-s001.zip › jimaging-4141588-supplementary.pdf]

Journal of Imaging

TABLE S1 : Consensus report.

## S1. Integrative discussion on the references process of inclusion: Consensus-Based Report

The consensus-based report (CR), prepared by A.L. and D.G., was used to verify the consistency between experiential insights collected in focus groups with RTTs and other MDT professionals and the findings from the narrative literature review. It served purely as a validation of alignment and internal consistency, ensuring that practice-informed observations were plausible and transferable across clinical settings.

The CR functioned as a validation step, ensuring that the proposed framework accurately reflects both published evidence and practical, real-world experience. It highlights the alignment between key RTT tasks, workflow optimization, and patient care priorities, distinguishing between current clinical practices and emerging applications such as AI-assisted tools and adaptive radiotherapy.

By combining convergent expert input with literature evidence, the CR strengthens the credibility, applicability, and transferability of the framework across different institutions and clinical settings.

**Table s1.** Consensus report.

| Reference | Type | Reason for inclusion | Alignment with focus group / CR validation |
|---|---|---|---|
| [1] | Pub | Provides epidemiological background on HN cancer risk factors relevant to MDT patient assessment and RTT awareness | Constructive for RTTs to understand risk factors; aligns with CR as fundamental context for patient monitoring and workflow |
| [2] | Pub | Updated review of HN cancer epidemiology informing clinical context | Supports RTTs in planning patient education and anticipating treatment complexity; validated in CR as consistent with experience |
| [3] | Pub | Overview of HN cancer pathophysiology and management | Highlights RTT role integration in multidisciplinary care; CR confirmed alignment with observed RTT contributions |
| [4] | Pub | Systematic review on MDT meetings' impact on outcomes | Reinforces value of structured MDTs; CR validated focus on workflow |

 https://doi.org/10.3390/jimaging12030117

| | | | |
|---|---|---|---|
| | | | coordination and early toxicity management |
| [5] | Pub | Evaluates value of HN tumor boards for staff and patients | Supports structured MDT participation; CR confirmed RTT observations on operational and collaborative benefits |
| [6] | Pub | Critical review of radiotherapy controversies in HN cancer | Provides context for emerging techniques and adaptive planning; validated in CR for consistency with expert experience |
| [7] | Pub | ESTRO benchmarking document for RTT postgraduate training | Guides alignment of proposed framework with international RTT competencies; CR confirmed relevance for training and skill development |
| [8] | Pub | Study on shoulder position variation and dosimetric impact | Important for patient positioning and immobilization accuracy; CR validation confirmed practical relevance |
| [9] | Pub | Effect of patient setup errors on IMRT SIB HN plans | Relevant for daily IGRT monitoring and QA; CR validated as consistent with practice-informed observations |
| [10] | Pub | Daily vs non-daily IGRT protocols in HN IMRT | Impacts workflow scheduling and imaging frequency; CR confirmed alignment with RTT daily tasks |
| [11] | Pub | Adequate setup margins and thresholds for immediate intervention | Supports safety checks and intervention criteria; CR validated as consistent with RTT vigilance practices |
| [12] | Pub | ESTRO ACROP guidelines for positioning and verification | Reference for immobilization protocols and QA; CR confirmed alignment with standardized practice |
| [13] | Pub | Open-face vs closed-face masks comparative study | Relevant for patient comfort, adherence, and adaptive planning; CR validated practical application in workflow |
| [14] | Pub | Early study on precise patient positioning | Provides historical context for immobilization practices; CR confirmed continued relevance for RTT reference |
| [15] | Pub | Setup accuracy of three thermoplastic masks | Reinforces current immobilization strategy; CR |

https://doi.org/10.3390/jimaging12030117

| | | | |
|---|---|---|---|
| | | | confirmed alignment with RTT focus on precision and reproducibility |
| [16] | Pub | Impact of patient anxiety on RT sessions | Highlights need for patient support and counseling; CR validated as consistent with RTT patient-centered activities |
| [17] | Pub | Mask anxiety management prospective study | Supports strategies to improve patient tolerance and adherence; CR confirmed practical relevance in HN RT |
| [18] | Pub | Scoping review on open-face masks | Enhances workflow and patient-centered care; CR validated alignment with RTT experience and daily practice |
| [19] | Pub | ESTRO core curricula update 2011 | Guides competency-based training; CR confirmed framework alignment with educational needs |
| [20] | Pub | Phase III trial comparing RT and chemoradiotherapy | Relevant for toxicity monitoring and adaptive interventions; CR validated as constructive for clinical workflow planning |
| [21] | Pub | Randomized study comparing chemoradiotherapy vs RT alone in advanced HN cancer | Provides context for treatment intensity and toxicity monitoring; CR validated as consistent with RTT clinical observations |
| [22] | Pub | Multidisciplinary approach update for squamous HN carcinoma | Supports MDT integration rationale; CR confirmed as aligned with focus group emphasis on collaboration |
| [23] | Pub/Website | ESTRO 3rd edition core curriculum for RTTs | Provides competency framework; CR confirmed alignment with educational standards and RTT skill development |
| [24] | Pub | RTT profession and new technology integration | Highlights adaptation to emerging tech and workflow optimization; CR validated as reflecting observed RTT practice |
| [25] | Pub | Systematic review on advanced practice roles of RTTs | Contextualizes role expansion; CR confirmed as constructive for defining operational tasks and training needs |

| | | | |
|---|---|---|---|
| [26] | Pub | Duration of RT linked to survival in HN cancer | Supports workflow and scheduling optimization; CR validated for relevance to minimizing treatment interruptions |
| [27] | Pub | Diagnostic delays in cancer | Provides rationale for timely interventions and workflow monitoring; CR confirmed as consistent with RTT patient-centered activities |
| [28] | Pub/Book Chapter | Radiotherapy scheduling principles | Guides practical workflow organization; CR validated as aligned with focus group discussions on operational efficiency |
| [29] | Pub | Impact of radiotherapy delays on outcomes | Supports scheduling and patient management strategies; CR confirmed alignment with RTT experience on timing adherence |
| [30] | Pub | Changes in pretreatment delays over a decade (DAHANCA survey) | Provides historical perspective on workflow improvement; CR validated as relevant for MDT optimization |
| [31] | Pub | Systematic review of waiting time and clinical outcomes | Informs RTT role in monitoring delays and patient scheduling; CR confirmed constructive for operational planning |
| [32] | Pub | Cost comparison and adaptive replanning in HN RT | Highlights economic and workflow considerations; CR validated as relevant for RTT contribution to adaptive planning |
| [33] | Pub | GORTEC practical and technical challenges in ART | Identifies key operational challenges; CR confirmed alignment with observed RTT tasks in adaptive radiotherapy |
| [34] | Pub | Dose variations in tumor volumes and OARs during IMRT | Supports importance of precise imaging and monitoring; CR validated as consistent with RTT focus on accuracy |
| [35] | Pub | Conceptual considerations for ART in HN cancer | Guides adaptive workflow design; CR confirmed alignment with practice-informed strategies |

https://doi.org/10.3390/jimaging12030117

| | | | |
|---|---|---|---|
| [36] | Pub | Triggered adaptive replanning in routine practice | Provides evidence for operational implementation; CR validated as consistent with RTT workflow observations |
| [37] | Pub | International survey on RTT education and training | Supports standardized curricula development; CR confirmed constructive for training alignment |
| [38] | Pub | Concordance between advanced practice RTTs and ROs in toxicity assessment | Reinforces RTT contribution to patient monitoring; CR validated as reflecting observed interprofessional collaboration |
| [39] | Pub | Multidisciplinary clinical pathway for dysphagia services | Demonstrates structured MDT impact on patient care; CR confirmed as aligned with RTT role in pathway implementation |
| [40] | Pub | Systematic review on advanced practice nursing impact | Provides parallel evidence for advanced practice roles and outcomes; CR validated as conceptually supportive for RTT operational development |
| [41] | Pub | Scoping review of established advanced practice roles in RT | Confirms operational tasks and scope; CR validated as reflecting observed RTT responsibilities |
| [42] | Pub | Role of practice standardization in outcome optimization | Provides rationale for workflow consistency; CR aligned with focus group discussions on operational coherence |
| [43] | Pub | MDT experience in HN cancer | Illustrates real-world MDT practices; CR validated as consistent with experiential focus group insights |
| [44] | Pub/Website | RTT advanced practice and future impact | Highlights potential and operational relevance; CR validated as aligned with focus group consensus on practice feasibility |
| [45] | Pub | Systematic review on clinical ART in HN cancer | Informs adaptive workflow implementation; CR confirmed constructive for daily RTT monitoring practices |
| [46] | Pub | Adaptive RT impact on dosimetric and clinical outcomes | Provides evidence for adaptive interventions; CR validated as consistent with |

https://doi.org/10.3390/jimaging12030117

| | | | |
|---|---|---|---|
| | | | RTT observations on workflow impact |
| [47] | Pub | Global advanced and extended radiographer practices | Contextualizes professional expansion internationally; CR confirmed alignment with observed advanced roles |
| [48] | Pub | AI in automating CT-based ART | Highlights emerging technologies; CR validated for separating routine vs experimental applications |
| [49] | Pub | Deep learning-based auto-segmentation in HN | Demonstrates AI tool application; CR confirmed constructive for training and clinical workflow relevance |
| [50] | Pub | General model for HN auto-segmentation with pre-treatment imaging | Supports integration of AI in planning; CR validated as aligned with RTT experience in adaptive workflows |
| [51] | Pub | AI role in clinical RT practice | Illustrates clinical translation of AI; CR confirmed as constructive for workflow integration |
| [52] | Website | Humanitas AI project for adaptive HN RT | Provides real-world AI application example; CR validated as supportive for patient-centered planning |
| [53] | Pub | Future of MDT in adaptive therapy | Contextualizes MDT evolution; CR confirmed as consistent with observed interprofessional collaboration |
| [54] | Website/Thesis | Clinical reasoning in RT | Supports decision-making frameworks; CR validated as relevant for RTT operational insights |
| [55] | Pub | Genome-based model for RT dose adjustment (GARD) | Provides precision RT rationale; CR validated as aligned with adaptive and individualized planning discussions |
| [56] | Pub | Pan-cancer prediction of RT benefit using genomic-adjusted dose | Supports evidence for predictive models; CR confirmed as constructive for potential workflow integration |
| [57] | Pub | Radiomics basics, methods, limitations | Provides theoretical foundation; CR validated as consistent with observed use of imaging data in practice |
| [58] | Pub | Radiomics and machine learning in HN RT | Highlights emerging precision tools; CR validated |

| | | | |
|---|---|---|---|
| | | | as constructive for future RTT workflow considerations |
| [59] | Pub | Systematic review on AI-enhanced RT workflow for HN cancer | Illustrates AI applications in workflow optimization; CR validated as constructive for distinguishing routine vs experimental use |
| [60] | Pub | Radiomics applications in HN tumor imaging: state of the art and challenges | Provides evidence on imaging-derived predictive features; CR confirmed as aligned with RTT observational insights |
| [61] | Pub | AI-driven radiomics in HN cancer: current status and future prospects | Highlights emerging precision tools; CR validated for consistency with focus group reflections on adaptive planning potential |
| [62] | Pub | Radiomics promises and challenges in HN radiotherapy | Contextualizes limitations and opportunities of radiomics; CR confirmed as constructive for clinical feasibility and training considerations |
| [63] | Pub | Radiotherapy and systemic therapies in HN cancer | Integrates multimodal treatment context; CR validated as supportive for MDT workflow and patient-centered planning alignment |

## References


1. Dhull, A.K.; Atri, R.; Dhankhar, R.; Chauhan, A.K.; Kaushal, V. Major Risk Factors in Head and Neck Cancer: A Retrospective Analysis of 12-Year Experiences. *World J. Oncol.* **2018**, *9*, 80–84. https://doi.org/10.14740/wjon1104w.
2. Aupérin, A. Epidemiology of head and neck cancers: An update. *Curr. Opin. Oncol.* **2020**, *32*, 178–186. https://doi.org/10.1097/CCO.0000000000000629.
3. Chow, L.Q.M. Head and Neck Cancer. *N. Engl. J. Med.* **2020**, *382*, 60–72. https://doi.org/10.1056/NEJMra1715715.
4. Pillay, B.; Wootten, A.C.; Crowe, H.; Corcoran, N.; Tran, B.; Bowden, P.; Crowe, J.; Costello, A.J. The impact of multidisciplinary team meetings on patient assessment, management and outcomes in oncology settings: A systematic review of the literature. *Cancer Treat. Rev.* **2016**, *42*, 56–72. https://doi.org/10.1016/j.ctrv.2015.11.007.
5. Westin, T.; Stalfors, J. Tumour boards/multidisciplinary head and neck cancer meetings: Are they of value to patients, treating staff or a political additional drain on healthcare resources? *Curr. Opin. Otolaryngol. Head Neck Surg.* **2008**, *16*, 103–107. https://doi.org/10.1097/MOO.0b013e3282f6a4c4.
6. De Felice, F.; Polimeni, A.; Valentini, V.; Brugnoletti, O.; Cassoni, A.; Greco, A.; de Vincentiis, M.; Tombolini, V. Radiotherapy Controversies and Prospective in Head and Neck Cancer: A Literature-Based Critical Review. *Neoplasia* **2018**, *20*, 227–232. https://doi.org/10.1016/j.neo.2018.01.002.
7. Coffey, M.; Leech, M.; ESTRO Radiation TherapisT Committee. The European Society of Radiotherapy and Oncology (ESTRO) European Higher Education Area levels 7 and 8 postgraduate benchmarking document for Radiation TherapisTs (RTTs). *Tech. Innov. Patient Support Radiat. Oncol.* **2018**, *8*, 22–40. https://doi.org/10.1016/j.tipsro.2018.09.009.

8. Neubauer, E.; Dong, L.; Followill, D.S.; Garden, A.S.; Court, L.E.; White, R.A.; Kry, S.F. Assessment of shoulder position variation and its impact on IMRT and VMAT doses for head and neck cancer. *Radiat. Oncol.* **2012**, *7*, 19. https://doi.org/10.1186/1748-717X-7-19.
9. Siebers, J.V.; Keall, P.J.; Wu, Q.; Williamson, J.F.; Schmidt-Ullrich, R.K. Effect of patient setup errors on simultaneously integrated boost head and neck IMRT treatment plans. *Int. J. Radiat. Oncol. Biol. Phys.* **2005**, *63*, 422–433. https://doi.org/10.1016/j.ijrobp.2005.02.029.
10. Yu, Y.; Michaud, A.L.; Sreeraman, R.; Liu, T.; Purdy, J.A.; Chen, A.M. Comparison of daily versus nondaily image-guided radiotherapy protocols for patients treated with intensity-modulated radiotherapy for head and neck cancer. *Head Neck* **2014**, *36*, 992–997. https://doi.org/10.1002/hed.23401.
11. Kapanen, M.; Laaksomaa, M.; Tulijoki, T.; Peltola, S.; Wigren, T.; Hyödynmaa, S.; Kellokumpu-Lehtinen, P.-L. Estimation of adequate setup margins and threshold for position errors requiring immediate attention in head and neck cancer radiotherapy based on 2D image guidance. *Radiat. Oncol.* **2013**, *8*, 212. https://doi.org/10.1186/1748-717X-8-212.
12. Leech, M.; Coffey, M.; Mast, M.; Moura, F.; Osztavics, A.; Pasini, D.; Vaandering, A. ESTRO ACROP guidelines for positioning, immobilisation and position verification of head and neck patients for radiation therapists. *Tech. Innov. Patient Support Radiat. Oncol.* **2017**, *1*, 1–7. https://doi.org/10.1016/j.tipsro.2016.12.001.
13. Mulla, Z.; Alwassia, R.K.; Senan, E.M.; Soaida, S.; Mohamed, A.A.M.A.; Almerdhemah, H.; Iqbal, H.A.; Muamenah, H.M. A comparative study between open-face and closed-face masks for head and neck cancer (HNC) in radiation therapy. *Rep. Pr. Oncol. Radiother.* **2020**, *25*, 382–388. https://doi.org/10.1016/j.rpor.2020.03.009.
14. Verhey, L.J.; Goitein, M.; McNulty, P.; Munzenrider, J.E.; Suit, H.D. Precise positioning of patients for radiation therapy. *Int. J. Radiat. Oncol. Biol. Phys.* **1982**, *8*, 289–294. https://doi.org/10.1016/0360-3016(82)90530-2.
15. Gilbeau, L.; Octave-Prignot, M.; Loncol, T.; Renard, L.; Scalliet, P.; Grégoire, V. Comparison of setup accuracy of three different thermoplastic masks for the treatment of brain and head and neck tumors. *Radiother. Oncol.* **2001**, *58*, 155–162. https://doi.org/10.1016/s0167-8140(00)00280-2.
16. Clover, K.; Oultram, S.; Adams, C.; Cross, L.; Findlay, N.; Ponman, L. Disruption to radiation therapy sessions due to anxiety among patients receiving radiation therapy to the head and neck area can be predicted using patient self-report measures. *Psycho-Oncology* **2011**, *20*, 1334–1341. https://doi.org/10.1002/pon.1854.
17. Nixon, J.L.; Brown, B.; Pigott, A.E.; Turner, J.; Brown, E.; Bernard, A.; Wall, L.R.; Ward, E.C.; Porceddu, S.V. A prospective examination of mask anxiety during radiotherapy for head and neck cancer and patient perceptions of management strategies. *J. Med. Radiat. Sci.* **2019**, *66*, 184–190. https://doi.org/10.1002/jmrs.346.
18. Lastrucci, A.; Morelli, I.; Votta, C.; Maran, I.; Iosca, N.; Monaco, I.P.; Salvestrini, V.; Desideri, I.; Marrazzo, L.; Wandael, Y.; et al. Open-Face Masks in Radiotherapy: Enhancing Therapeutic Strategies for Head and Neck and Brain Cancer Patients—A Comprehensive Scoping Review. *Cancers* **2024**, *16*, 2899. https://doi.org/10.3390/cancers16162899.
19. Eriksen, J.G.; Beavis, A.W.; Coffey, M.A.; Leer, J.W.H.; Magrini, S.M.; Benstead, K.; Boelling, T.; Hjälm-Eriksson, M.; Kantor, G.; Maciejewski, B.; et al. The updated ESTRO core curricula 2011 for clinicians, medical physicists and RTTs in radiotherapy/radiation oncology. *Radiother. Oncol.* **2012**, *103*, 103–108. https://doi.org/10.1016/j.radonc.2012.02.007.
20. Adelstein, D.J.; Li, Y.; Adams, G.L.; Wagner, H., Jr.; Kish, J.A.; Ensley, J.F.; Schuller, D.E.; Forastiere, A.A. An intergroup phase III comparison of standard radiation therapy and two schedules of concurrent chemoradiotherapy in patients with unresectable squamous cell head and neck cancer. *J. Clin. Oncol.* **2003**, *21*, 92–98. https://doi.org/10.1200/jco.2003.01.008.
21. Wendt, T.G.; Grabenbauer, G.G.; Rödel, C.M.; Thiel, H.J.; Aydin, H.; Rohloff, R.; Wustrow, T.P.; Iro, H.; Popella, C.; Schalhorn, A. Simultaneous radiochemotherapy versus radiotherapy alone in advanced head and neck cancer: A randomized multicenter study. *J. Clin. Oncol.* **1998**, *16*, 1318–1324. https://doi.org/10.1200/jco.1998.16.4.1318.
22. Bernier, J. A multidisciplinary approach to squamous cell carcinomas of the head and neck: An update. *Curr. Opin. Oncol.* **2008**, *20*, 249–255. https://doi.org/10.1097/cco.0b013e3282faa0b1.
23. Coffeya, M.A.; Mullaneyb, L.; Bojenc, A.; Vaanderingd, A.; Vandeveldee, G. Recommended ESTRO core curriculum for RTTs (Radiation Therapists)–III Edition. Available online: https://www.estro.org/binaries/content/assets/estro/school/european-curricula/recommended_core_curriculum-radiationtherapists-3rd-edition-2011.pdf (accessed on 1 April 2025 ).
24. Leech, M.; Abdalqader, A.; Alexander, S.; Anderson, N.; Barbosa, B.; Callens, D.; Chapman, V.; Coffey, M.; Cox, M.; Curic, I.; et al. The Radiation Therapist profession through the lens of new technology: A practice development paper based on the ESTRO Radiation Therapist Workshops. *Tech. Innov. Patient Support Radiat. Oncol.* **2024**, *30*, 100243. https://doi.org/10.1016/j.tipsro.2024.100243.

25. Oliveira, C.; Barbosa, B.; Couto, J.; Bravo, I.; Khine, R.; McNair, H. Advanced practice roles of therapeutic radiographers/radiation therapists: A systematic literature review. *Radiography* **2022**, *28*, 605–619. https://doi.org/10.1016/j.radi.2022.04.009.
26. Mazul, A.L.; Stepan, K.O.; Barrett, T.F.; Thorstad, W.L.; Massa, S.; Adkins, D.R.; Daly, M.D.; Rich, J.T.; Paniello, R.C.; Pipkorn, P.; et al. Duration of radiation therapy is associated with worse survival in head and neck cancer. *Oral Oncol.* **2020**, *108*, 104819. https://doi.org/10.1016/j.oraloncology.2020.104819.
27. Neal, R.D. Do diagnostic delays in cancer matter? *Br. J. Cancer* **2009**, *101* (Suppl. S2), S9–S12. https://doi.org/10.1038/sj.bjc.6605384.
28. Petrovic, D.; Castro, E.; Petrovic, S.; Kapamara, T. Radiotherapy Scheduling. In *Automated Scheduling and Planning;* Uyar, A.S., Ozcan, E., Urquhart, N., Eds.; Studies in Computational Intelligence; Springer: Berlin, Germany; Heidelberg, Germany; 2013; Volume 505, pp. 155–189. https://doi.org/10.1007/978-3-642-39304-4_7.
29. Žumer, B.; Perme, M.P.; Jereb, S.; Strojan, P. Impact of delays in radiotherapy of head and neck cancer on outcome. *Radiat. Oncol.* **2020**, *15*, 202. https://doi.org/10.1186/s13014-020-01645-w.
30. Primdahl, H.; Nielsen, A.L.; Larsen, S.; Andersen, E.; Ipsen, M.; Lajer, C.; Vestermark, L.W.; Andersen, L.J.; Hansen, H.S.; Overgaard, M.; et al. Changes from 1992 to 2002 in the pretreatment delay for patients with squamous cell carcinoma of larynx or pharynx: A Danish nationwide survey from DAHANCA. *Acta Oncol.* **2006**, *45*, 156–161. https://doi.org/10.1080/02841860500423948.
31. Chen, Z.; King, W.; Pearcey, R.; Kerba, M.; Mackillop, W.J. The relationship between waiting time for radiotherapy and clinical outcomes: A systematic review of the literature. *Radiother. Oncol.* **2008**, *87*, 3–16. https://doi.org/10.1016/j.radonc.2007.11.016.
32. Walls, R.; McLaughlin, C. Comparison and Breakdown of Cost in Head and Neck Cancer Radiation Therapy and Adaptive Replanning. *Int. J. Radiat. Oncol.* **2023**, *117*, e632. https://doi.org/10.1016/j.ijrobp.2023.06.2029.
33. Delaby, N.; Barateau, A.; Chiavassa, S.; Biston, M.-C.; Chartier, P.; Graulières, E.; Guinement, L.; Huger, S.; Lacornerie, T.; Millardet-Martin, C.; et al. Practical and technical key challenges in head and neck adaptive radiotherapy: The GORTEC point of view. *Phys. Medica* **2023**, *109*, 102568. https://doi.org/10.1016/j.ejmp.2023.102568.
34. Beltran, M.; Ramos, M.; Rovira, J.J.; Perez-Hoyos, S.; Sancho, M.; Puertas, E.; Benavente, S.; Ginjaume, M.; Giralt, J. Dose variations in tumor volumes and organs at risk during IMRT for head-and-neck cancer. *J. Appl. Clin. Med. Phys.* **2012**, *13*, 3723. https://doi.org/10.1120/jacmp.v13i6.3723.
35. Heukelom, J.; Fuller, C.D. Head and Neck Cancer Adaptive Radiation Therapy (ART): Conceptual Considerations for the Informed Clinician. *Semin. Radiat. Oncol.* **2019**, *29*, 258–273. https://doi.org/10.1016/j.semradonc.2019.02.008.
36. Figen, M.; Öksüz, D.Ç.; Duman, E.; Prestwich, R.; Dyker, K.; Cardale, K.; Ramasamy, S.; Murray, P.; Şen, M. Radiotherapy for Head and Neck Cancer: Evaluation of Triggered Adaptive Replanning in Routine Practice. *Front. Oncol.* **2020**, *10*, 579917. https://doi.org/10.3389/fonc.2020.579917.
37. Leech, M.; Coffey, M.; Jeha, J.; Ben Prajogi, G.; Bakhishova, K.; Wakeham, K. Radiation Therapist Education and Training: An International Survey. *JCO Glob. Oncol.* **2024**, *10*, e2300317. https://doi.org/10.1200/go.23.00317.
38. Sin, S.; Chua, M.L.; Wong, S.M.; Sommat, K.; Lin, X.; Ng, Y.; Soong, Y. An evaluation of concordance between head and neck advanced practice radiation therapist and radiation oncologists in toxicity assessment for nasopharyngeal carcinoma patients. *Tech. Innov. Patient Support Radiat. Oncol.* **2021**, *19*, 52–56. https://doi.org/10.1016/j.tipsro.2021.08.001.
39. Messing, B.P.; Ward, E.C.; Lazarus, C.; Ryniak, K.; Kim, M.; Silinonte, J.; Gold, D.; Thompson, C.B.; Pitman, K.T.; Blanco, R.; et al. Establishing a Multidisciplinary Head and Neck Clinical Pathway: An Implementation Evaluation and Audit of Dysphagia-Related Services and Outcomes. *Dysphagia* **2019**, *34*, 89–104. https://doi.org/10.1007/s00455-018-9917-4.
40. Woo, B.F.Y.; Lee, J.X.Y.; Tam, W.W.S. The impact of the advanced practice nursing role on quality of care, clinical outcomes, patient satisfaction, and cost in the emergency and critical care settings: A systematic review. *Hum. Resour. Health* **2017**, *15*, 63. https://doi.org/10.1186/s12960-017-0237-9.
41. Lawlor, S.; Leech, M. Established advanced practice roles in radiation therapy: A scoping review. *J. Med Imaging Radiat. Oncol.* **2024**, *68*, 342–352. https://doi.org/10.1111/1754-9485.13634.
42. Dimmer, A.; Baird, R.; Puligandla, P. Role of practice standardization in outcome optimization for CDH. *World J. Pediatr. Surg.* **2024**, *7*, e000783. https://doi.org/10.1136/wjps-2024-000783.
43. Prgomet, D.; Bišof, V.; Prstačić, R.; Radivojević, R.C.; Brajković, L.; Šimić, I. The Multidisciplinary Team (MDT) in the Treatment of Head and Neck Cancer–A Single-institution Experience. *Acta Clin. Croat.* **2022**, *61*, 77–87. https://doi.org/10.20471/acc.2022.61.s4.10.

44. Duffton, A.; Harnett, N.; McNair, H.A.; Bennett, E.; Clarkson, M.; Couto, J.G.; Lawler, G.; Matthews, K.; Oliveira, C.; Rozanec, N.; et al. RTT advanced practice and how it can change the future of radiotherapy. *Tech. Innov. Patient Support Radiat. Oncol.* **2024**, *30*, 100245. https://doi.org/10.1016/j.tipsro.2024.100245.
45. Lindegaard, A.M.; Håkansson, K.; Bernsdorf, M.; Gothelf, A.B.; Kristensen, C.A.; Specht, L.; Vogelius, I.R.; Friborg, J. A systematic review on clinical adaptive radiotherapy for head and neck cancer. *Acta Oncol.* **2023**, *62*, 1360–1368. https://doi.org/10.1080/0284186x.2023.2245555.
46. Simopoulou, F.M.; Kyrgias, G.; Georgakopoulos, I.; Avgousti, R.M.; Armpilia, C.M.; Skarlos, P.; Softa, V.M.; Theodorou, K.M.; Kouloulias, V.; Zygogianni, A. Does adaptive radiotherapy for head and neck cancer favorably impact dosimetric, clinical, and toxicity outcomes?: A review. *Medicine* **2024**, *103*, e38529. https://doi.org/10.1097/md.0000000000038529.
47. Carlier, S.; Mendes, L.F.; Lago, L.P.; Ding, S. The extended and advanced clinical practices of radiographers worldwide: A scoping review. *J. Med. Imaging Radiat. Sci.* **2025**, *56*, 101818. https://doi.org/10.1016/j.jmir.2024.101818.
48. Mastella, E.; Calderoni, F.; Manco, L.; Ferioli, M.; Medoro, S.; Turra, A.; Giganti, M.; Stefanelli, A. A systematic review of the role of artificial intelligence in automating computed tomography-based adaptive radiotherapy for head and neck cancer. *Phys. Imaging Radiat. Oncol.* **2025**, *33*, 100731. https://doi.org/10.1016/j.phro.2025.100731.
49. Kawahara, D.; Tsuneda, M.; Ozawa, S.; Okamoto, H.; Nakamura, M.; Nishio, T.; Nagata, Y. Deep learning-based auto segmentation using generative adversarial network on magnetic resonance images obtained for head and neck cancer patients. *J. Appl. Clin. Med. Phys.* **2022**, *23*, e13579. https://doi.org/10.1002/acm2.13579.
50. Clark, B.; Hardcastle, N.; Gaudreault, M.; Johnston, L.A.; Korte, J.C. A general model for head and neck auto-segmentation with patient pre-treatment imaging during adaptive radiation therapy. *Med. Phys.* **2025**, *52*, 4590–4597. https://doi.org/10.1002/mp.17732.
51. Kawamura, M.; Kamomae, T.; Yanagawa, M.; Kamagata, K.; Fujita, S.; Ueda, D.; Matsui, Y.; Fushimi, Y.; Fujioka, T.; Nozaki, T.; et al. Revolutionizing radiation therapy: The role of AI in clinical practice. *J. Radiat. Res.* **2024**, *65*, 1–9. https://doi.org/10.1093/jrr/rrad090.
52. Available online: https://www.humanitas.eu/projects/artificial-intelligence-for-locally-advanced-head-and-neck-cancer-treated-with-multi-modality-adaptive-radiotherapy-machine-learning-based-radiomic-prediction-of-outcome-and-toxicity-radiomicart (accessed on 1 April 2025).
53. McNair, H.; Milosevic, M.; Parikh, P.; van der Heide, U. Future of Multidisciplinary Team in the Context of Adaptive Therapy. *Semin. Radiat. Oncol.* **2024**, *34*, 418–425. https://doi.org/10.1016/j.semradonc.2024.08.006.
54. Collins, M. (2018). Clinical reasoning in image guided radiotherapy: A multimethod study. https://doi.org/10.7190/shu-thesis-00124 Available online: https://shura.shu.ac.uk/23419/1/Collins_2018_ProfD_ClinicalReasoningIn.pdf (accessed on 10 January 2026).
55. Scott, J.G.; Berglund, A.; Schell, M.J.; Mihaylov, I.; Fulp, W.J.; Yue, B.; Welsh, E.; Caudell, J.J.; Ahmed, K.; Strom, T.S.; et al. A genome-based model for adjusting radiotherapy dose (GARD): A retrospective, cohort-based study. *Lancet Oncol.* **2017**, *18*, 202–211. https://doi.org/10.1016/s1470-2045(16)30648-9.
56. Scott, J. G., Sedor, G., Ellsworth, P., Scarborough, J. A., Ahmed, K. A., Oliver, D. E., Eschrich, S. A., Kattan, M. W., & Torres-Roca, J. F. (2021). Pan-cancer prediction of radiotherapy benefit using genomic-adjusted radiation dose (GARD): a cohort-based pooled analysis. The Lancet. Oncology, 22(9), 1221–1229. https://doi.org/10.1016/S1470-2045(21)00347-8
57. Lohmann, P., Bousabarah, K., Hoevels, M., & Treuer, H. (2020). Radiomics in radiation oncology-basics, methods, and limitations. Strahlentherapie und Onkologie : Organ der Deutschen Rontgengesellschaft ... [et al], 196(10), 848–855. https://doi.org/10.1007/s00066-020-01663-3
58. Giraud, P., Giraud, P., Gasnier, A., El Ayachy, R., Kreps, S., Foy, J. P., Durdux, C., Huguet, F., Burgun, A., & Bibault, J. E. (2019). Radiomics and Machine Learning for Radiotherapy in Head and Neck Cancers. Frontiers in oncology, 9, 174. https://doi.org/10.3389/fonc.2019.00174
59. Franzese, C., Dei, D., Lambri, N., Teriaca, M. A., Badalamenti, M., Crespi, L., Tomatis, S., Loiacono, D., Mancosu, P., & Scorsetti, M. (2023). Enhancing Radiotherapy Workflow for Head and Neck Cancer with Artificial Intelligence: A Systematic Review. Journal of personalized medicine, 13(6), 946. https://doi.org/10.3390/jpm13060946
60. Tortora M, Gemini L, Scaravilli A, Ugga L, Ponsiglione A, Stanzione A, D'Arco F, D'Anna G, Cuocolo R. Radiomics Applications in Head and Neck Tumor Imaging: A Narrative Review. Cancers (Basel). 2023 Feb 12;15(4):1174. doi: 10.3390/cancers15041174. PMID: 36831517; PMCID: PMC9954362.

61. Alabi, R. O., Elmusrati, M., Leivo, I., Almangush, A., & Mäkitie, A. A. (2024). Artificial Intelligence-Driven Radiomics in Head and Neck Cancer: Current Status and Future Prospects. International journal of medical informatics, 188, 105464. https://doi.org/10.1016/j.ijmedinf.2024.105464.
62. Iancu, R. I., Zara, A. D., Mirestean, C. C., & Iancu, D. P. T. (2021). Radiomics in Head and Neck Cancers Radiotherapy. Promises and Challenges. Maedica, 16(3), 482–488. https://doi.org/10.26574/maedica.2020.16.3.482
63. De Felice, F.; Cattaneo, C.G.; Franco, P. Radiotherapy and Systemic Therapies: Focus on Head and Neck Cancer. *Cancers* **2023**, *15*, 4232. https://doi.org/10.3390/cancers15174232.


**Disclaimer/Publisher's Note:** The statements, opinions and data contained in all publications are solely those of the individual author(s) and contributor(s) and not of MDPI and/or the editor(s). MDPI and/or the editor(s) disclaim responsibility for any injury to people or property resulting from any ideas, methods, instructions or products referred to in the content.
